# PIEZO1 acts as a cancer suppressor by regulating the ROS/Wnt/β‐catenin axis

**DOI:** 10.1111/1759-7714.15278

**Published:** 2024-03-18

**Authors:** Haimei Bo, Qi Wu, Chaonan Zhu, Yang Zheng, Guang Cheng, Lihua Cui

**Affiliations:** ^1^ Tianjin Medical University General Hospital Tianjin China; ^2^ North China University of Science and Technology Tangshan China; ^3^ Graduate School Tianjin Medical University Tianjin China

**Keywords:** EMT, lung adenocarcinoma, PIEZO1, ROS, Wnt/β‐catenin

## Abstract

**Background:**

PIEZO1 works differently in different cancers and at different stages of development. The objective of the current study was to explore the function and underlying mechanism of PIEZO1 in lung adenocarcinoma (LUAD) cells.

**Methods:**

Different LUAD cell lines were treated with PIEZO1 inhibitor (GsMTx4) and agonist (Yoda1), and the expression of PIEZO1 in LUAD cells was detected using real‐time quantitative PCR (RT‐qPCR) and western blotting. The effects of PIEZO1 on invasion, migration and epithelial‐mesenchymal transition (EMT) markers protein expression of LUAD cells were detected using the MTT assay, flow cytometry, transwell assay, wound‐healing assay, and western blotting. Reactive oxygen species (ROS) agonists (BAY 87–2243) and inhibitors (NAC) and Wnt/β‐catenin pathway inhibitors (iCRT3) were selected to treat A549 cells to investigate the mechanism of PIEZO1 on ROS production and Wnt/β‐catenin expression in A549 cells.

**Results:**

In A549, NCI‐H1395, and NCI‐H1975 cells, GsMTx4 promoted cell proliferation, invasion, migration, upregulated EMT‐related marker protein expression, and inhibited cell apoptosis, while Yoda1 exerted effects opposite to those of GsMTx4. In A549 cells, GsMTx4 can reduce ROS production, it also inhibited ROS production, apoptosis, and downregulated proapoptotic markers induced by BAY 87–2243. Importantly, BAY 87–2243 blocked the effect of GSMTX4‐induced Wnt/β‐catenin overexpression. Similarly, Yoda1 can reduce the effect of NAC. In addition, iCRT3 can block the upregulation of EMT‐related marker proteins by GsMTx4, and increase apoptosis and decrease cell invasion and migration.

**Conclusion:**

In summary, PIEZO1 acts as a cancer suppressor by regulating the ROS/Wnt/β‐catenin axis, providing a new perspective on the role of mechanosensitive channel proteins in cancer.

## Graphical abstract

Treatment with PIEZO1 inhibitors decreased the expression of PIEZO1 in A549 cells, and low levels of PIEZO1 promoted the activation of the Wnt/β‐catenin pathway by decreasing ROS levels, thus promoting A549 cell proliferation, invasion, and EMT.

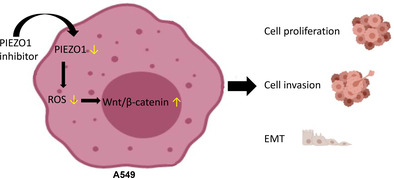

## INTRODUCTION

Regarded as one of the most common malignancies, lung cancer is the leading cause of cancer‐related deaths globally.^1^ Lung adenocarcinoma (LUAD) is the most common type of lung cancer.^2^ Despite the emergence and development of new therapies in recent years, LUAD remains an aggressive and rapidly lethal cancer type.^3^ In 2022, lung cancer was among the top three most common cancers in males and females.^4^ Therefore, exploring the molecular mechanisms underlying LUAD progression is crucial to identify new therapeutic targets.

Epithelial‐to‐mesenchymal transition (EMT) is a major driver of cancer progression.^5^ Recent studies have shown that EMT promotes the invasive and metastatic abilities of lung cancer.^6^, ^7^ PIEZO proteins are ion channels that mediate cellular mechanosensory transduction, including PIEZO1 and PIEZO2.^8^ PIEZO protein is a novel mechanosensitive cation channel protein discovered in a mouse neuroblastoma cell line in 2010.^9^ As a member of the PIEZO family, PIEZO1 reportedly participates in various physiological processes, including sensing blood flow and shear stress to promote vascular development, modulating red blood cell function, and regulating cell migration and differentiation.^10^ The role of PIEZO1 in tumor invasion and metastasis has received growing attention. PIEZO1 knockdown was shown to promote breast cancer cell migration and invasion by weakening cellular mechanical properties and inhibiting matrix metalloproteinase expression.^11^ Meanwhile, overexpression of PIEZO1 can promote the migration and motility of colon cancer cells via a potential mechanism related to the PIEZO1‐MCU‐HIF‐1α‐VEGF axis.^12^ In lung cancer, the function of PIEZO1 has been linked to disease development. PIEZO was found to be downregulated in non‐small cell lung cancer (NSCLC) tumor tissues, and the absence of PIEZO1 could substantially promote NSCLC cell migration in vitro and tumor growth in vivo^13^; however, the underlying mechanism remains unclear. Zhang et al. have shown that the expression of Piezo1 was time‐dependently increased in A549 cells after cyclic stretching.^14^ Currently, studies on Piezo1 in NCI‐H1975 cells are lacking.

Herein, we examined the effects of PIEZO1 on the biological behavior of LUAD cells using GsMTx4 (a PIEZO1 inhibitor) and Yoda1 (a PIEZO1 agonist).^15^, ^16^ We explored the PIEZO1‐mediated mechanisms underlying the regulation of proliferation and apoptosis in A549 cells. Reactive oxygen species (ROS) are a class of one‐electron reduction products of oxygen in vivo that participate in various physiological processes.^17^, ^18^ BAY 87‐2243 has been shown to promote ROS production.^19^ To explore whether PIEZO1 modulates the EMT process in A549 cells through the Wnt/β‐catenin pathway, iCRT3 (a β‐catenin inhibitor) was employed.^20^ Accordingly, we aimed to investigate the role of PIEZO1 in LUAD progression and determine its potential molecular mechanisms to provide new insights into PIEZO1‐based targeted therapies.

## METHODS

### Cell culture

A549 cells were cultured in F12K medium (Gibco) supplemented with 10% fetal bovine serum (FBS; Gibco). NCI‐H1395 and NCI‐H1975 cells were cultured in RPMI‐1640 medium (Gibco) containing 10% FBS. Cells were cultured in a humidified incubator with 5% CO_2_ at 37°C. All cell lines were obtained from the Cell Bank of the Chinese Academy of Sciences (Shanghai, China).

### Methyl thiazolyl tetrazolium assay (MTT)

The MTT assay was used to measure cell proliferation. Briefly, cells were collected and seeded into 96‐well plates, with 3 × 10^3^ cells/well, and incubated overnight. The MTT reagent (5 mg/mL, Sigma) was added to each well and incubated for 4 h. The supernatant was removed, and 150 μL of dimethyl sulfoxide (DMSO) was added to each well and shaken at low speed for 10 min to dissolve crystals. The absorbance was measured at 490 nm using a microplate reader (Hangzhou Allsheng Instruments Co. Ltd).

### Flow cytometry

The apoptosis rate was detected using an annexinV‐FITC/PI apoptosis detection kit (Becton Dickinson). Briefly, 1 × 10^6^ cells were centrifuged at 400 *× g* for 5 min and washed with ice‐cold phosphate‐buffered saline (PBS). Cells were then resuspended in 200 μL PBS, 10 μL annexin V‐FITC, and 10 μL PI and incubated at 4°C for 30 min in the dark. Apoptosis was detected by flow cytometry (ACEA Biosciences).

Intracellular ROS levels were measured using a ROS detection kit (Beyotime). DCFH‐DA was diluted with serum‐free culture medium at 1:1000 to attain a final concentration of 10 μM. Cells were collected and suspended in 1 mL of well‐diluted DCFH‐DA at a cell concentration of 1 × 10^6^/mL and incubated for 20 min. The prepared cell specimens were mixed by turning them upside down every 3 min to ensure full contact between the probe and the cells. Then, cells were washed three times with serum‐free cell culture medium. The cells were resuspended with 500 μL PBS. Finally, the cell‐related average fluorescence intensity was measured using flow cytometry, and the results were analyzed using NovoCyte software.

### Transwell invasion assay

Cell invasion ability was investigated using a Transwell chamber with a Matrigel matrix (BD). Formaldehyde (Sinopharm Chemical Reagent Co.) was added, and cells were fixed at room temperature for 20 min. Next, the cells were washed with PBS and stained with crystal violet solution (Solarbio) for 30 min. Cells in each field of view were counted under a 200× microscope.

### Wound‐healing assay

Cells were seeded at a density of 1 × 10^6^ cells in a six‐well plate the day before the experiment. On reaching nearly 100% confluency, cells were scratched with a p200 pipette tip. The cells were cultured in a serum‐free medium. Photographs were taken using a microscope at 0 and 24 h to observe scratch spacing.

### Western blot

Cells were lysed with RIPA buffer (Solarbio) to isolate the total protein. Protein concentrations were determined using a BCA kit (Solarbio). Subsequently, 20 μg of protein was achieved by performing sodium dodecyl sulfate‐polyacrylamide gel electrophoresis (SDS‐PAGE), and specimens were transferred onto polyvinylidene fluoride (PVDF) membranes (Millipore). Membranes were blocked in 5% skim milk for 2 h and then incubated with primary antibodies against Snail, vimentin, N‐cadherin, E‐cadherin, Wnt3a, and β‐catenin (1:1000; Bioswamp) at 4°C overnight. Secondary antibodies were then incubated at room temperature for 1 h. The signal was visualized using an ECL detection reagent (Millipore) and quantified using TANON GIS software.

### Reverse transcription‐quantitative polymerase chain reaction (RT‐qPCR)

Total RNA was extracted using the TRIzol reagent and reverse‐transcribed into cDNA using a reverse transcription kit (TAKARA, Japan). RT‐qPCR was performed using the SYBR FAST qPCR Master Mix in a CFX‐Connect 96 Real‐Time PCR System (Bio‐Rad, USA). GAPDH was used as an internal reference gene. The relative expression of PIEZO1 was calculated using the 2^−ΔΔCt^ method. Primer sequences were as follows: PIEZO1 forward primer, 5′‐TCTTCCTTAGCCATTACTACCT‐3′, PIEZO1 reverse primer, 5′‐TACGCTCCATCTGTCTTTTC‐3′. GAPDH forward primer, 5′‐GGGAAACTGTGGCGTGAT‐3′, GAPDH reverse primer, 5′‐GAGTGGGTGTCGCTGTTGA‐3′.

### Statistical analysis

Statistical analysis was performed using SPSS 19.0 (IBM Corporation). Data are expressed as mean ± standard deviation (SD). Comparisons between groups were performed using a one‐way analysis of variance (ANOVA) and LSD‐*t* test was used for post hoc analysis. *p < 0.05 indicated statistical significance.

## RESULTS

### Effect of PIEZO1 on the biological behavior of LUAD cells

**FIGURE 1:**
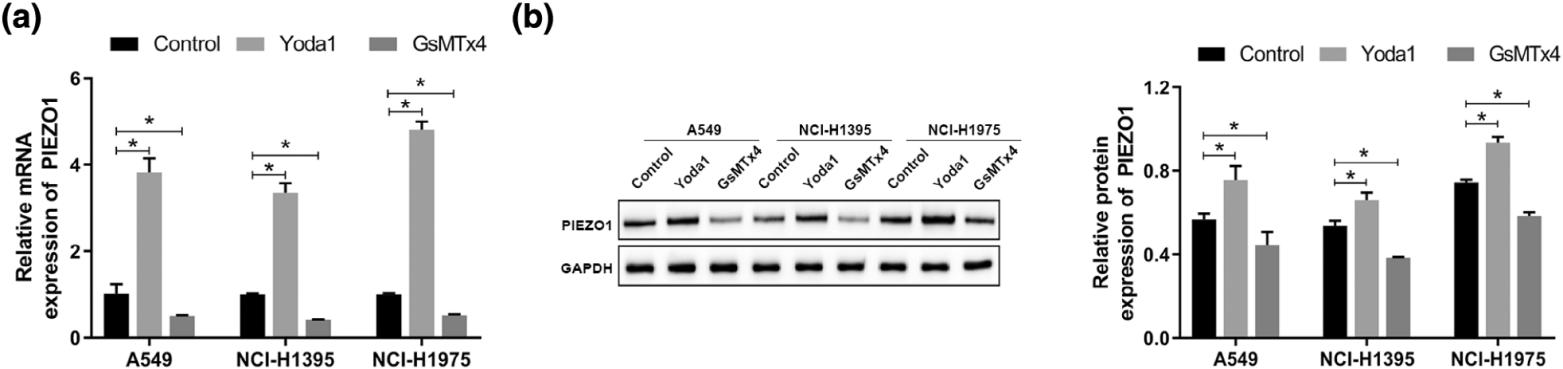
Effects of GsMTx4 and Yoda1 on PIEZO1 mRNA and protein expression in LUAD cells. (a) mRNA expression of PIEZO1 in LUAD cells was detected by qRT‐PCR. (b) The protein expression of PIEZO1 in LUAD cells was detected by Western blot. Data are presented as the mean ± standard deviation (SD). *p < 0.05. LUAD, lung adenocarcinoma; RT‐qPCR, reverse transcription‐quantitative PCR.

**FIGURE 2:**
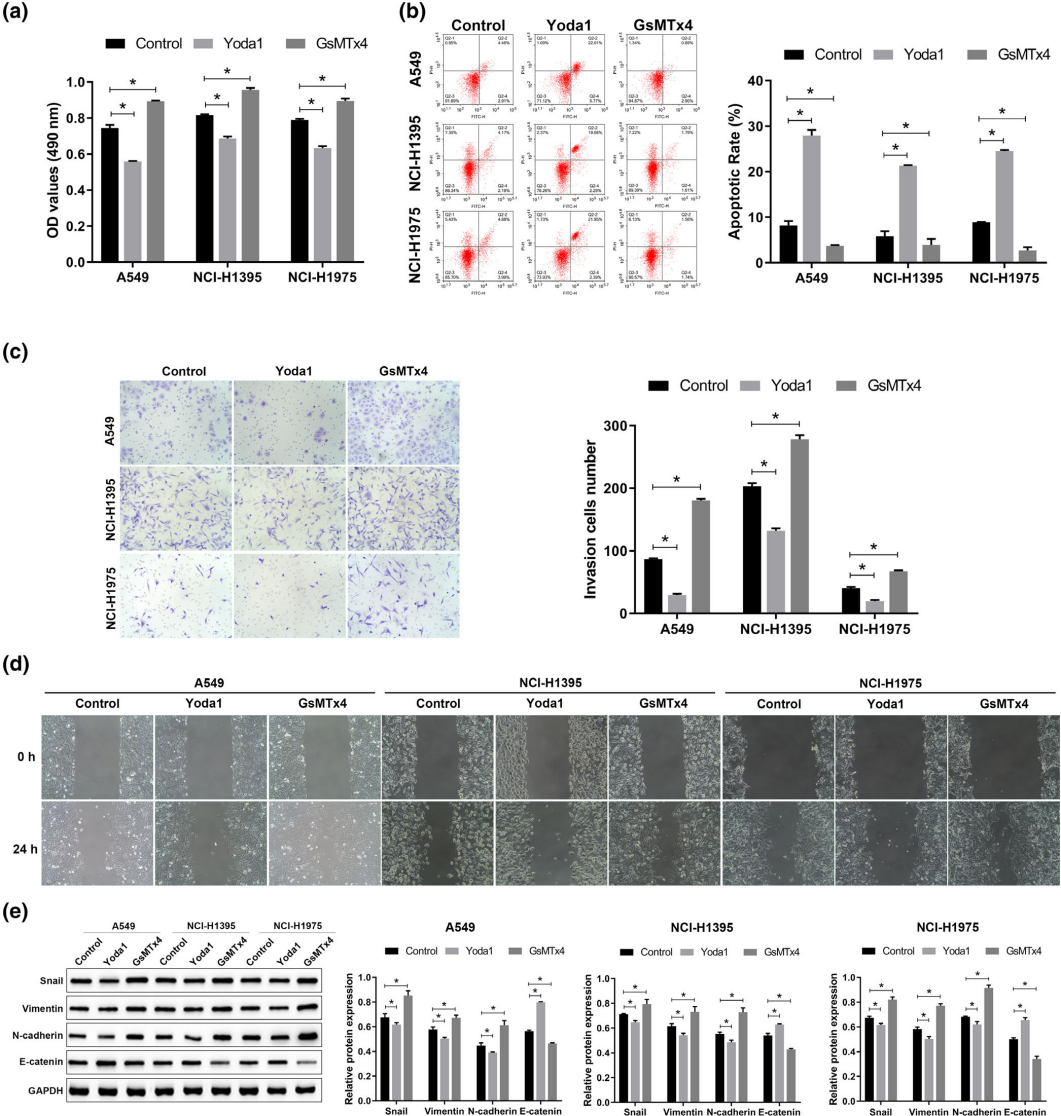
Effect of PIEZO1 on proliferation, apoptosis, invasion, migration, and EMT‐related protein expression of LUAD cells. (a) Cell viability of LUAD cells was detected using the methyl thiazolyl tetrazolium assay (MTT) assay. (b) The apoptosis rate of LUAD cells was detected by flow cytometry. (c) Cell invasion was evaluated by transwell assay (×200). (d) Cell migration was evaluated by performing the wound‐healing assay (×100). (f) The expressions of EMT‐related protein were detected by western blotting. Data are presented as the mean ± standard deviation (SD). *p < 0.05. EMT, epithelial‐mesenchymal transition; LUAD, lung adenocarcinoma.

First, we examined the effects of Yoda1 (a PIEZO1 activator) and GsMTx4 (a PIEZO1 inhibitor) on the mRNA and protein expression of PIEZO1 in three different LUAD cell lines: A549, NCI‐H1395, and NCI‐H1975. The results revealed that treatment with GsMTx4 decreased PIEZO1 expression in A549, NCI‐H1395, and NCI‐H1975 cells, whereas treatment with Yoda1 could induce PIEZO1 expression (Figure 1). To clarify the effects of PIEZO1 on the proliferation, apoptosis, invasion, and migration of LUAD cells, we treated A549, NCI‐H1395, and NCI‐H1975 cells with Yoda1, a PIEZO1 agonist, and GsMTx4, a PIEZO1 antagonist. We observed that treatment with GsMTx4 could promote proliferation, invasion, and migration and suppress apoptosis in LUAD cells, whereas treatment with Yoda1 inhibited proliferation, invasion, and migration and promoted apoptosis in LUAD cells (Figure 2a–d). We also examined the expression of EMT‐related proteins. Treatment with GsMTx4 upregulated the expression of Snail, vimentin, and N‐cadherin and downregulated that of E‐cadherin. Conversely, treatment with Yoda1 induced a phenomenon opposite to that associated with GsMTx4 treatment (Figure 2e). These results confirm that PIEZO1 plays an important role in LUAD progression. In addition, the proliferative capacity of A549 cells after Yoda1 induction was lower than that of NCI‐H1395 and NCI‐H1975 cells, and the apoptotic rate was higher than that of NCI‐H1395 and NCI‐H1975 cells, indicating that PIEZO1 had the greatest effect on A549 cells among the three cell lines examined. Therefore, A549 cells were selected for subsequent experiments.

### PIEZO1 affects the Wnt/β‐catenin pathway by regulating ROS levels in A549 cells

**FIGURE 3:**
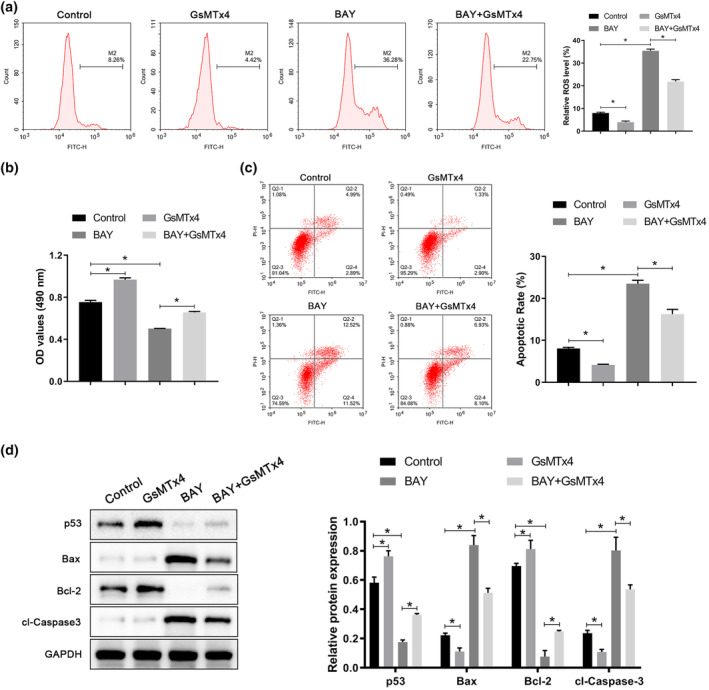
PIEZO1 regulates cell proliferation and apoptosis by regulating ROS levels in A549 cells. (a) ROS level in A549 cells was detected by flow cytometry. (b) Cell viability of A549 cells was detected by methyl thiazolyl tetrazolium assay (MTT) assay. (c) The apoptosis rate of A549 cells was detected by flow cytometry. (d) Expressions of p53, Bax, Bcl‐2, and cl‐caspase 3 protein were detected by western blotting. Data are presented as the mean ± standard deviation (SD). *p < 0.05. ROS, reactive oxygen species.

To clarify the regulatory effect of PIEZO1 on ROS, A549 cells were treated with GsMTx4 (PIEZO1 inhibitor) and BAY 87‐2243 (ROS agonist). We observed that treatment with GsMTx4 reversed the promotion of intracellular ROS by BAY 87‐2243 (Figure 3a). By regulating ROS, we further examined the effects of PIEZO1 on A549 cell proliferation and apoptosis. We found that treatment with GsMTx4 could reverse the effect of BAY 87‐2243 on the inhibition of proliferation and promotion of apoptosis in A549 cells (Figure 3b,c). Furthermore, we detected the expression of apoptosis‐related proteins, and the results revealed that treatment with BAY 87‐2243 could increase the expression levels of p53 and bcl‐2 proteins, decrease the expression levels of Bax and cl‐caspase‐3 proteins; treatment with GsMTx4 treatment could reverse these BAY 87‐2243‐mediated effects (Figure 3d). Collectively, these results suggest that PIEZO1 influences the proliferation and apoptosis of A549 cells by regulating ROS levels.

**FIGURE 4:**
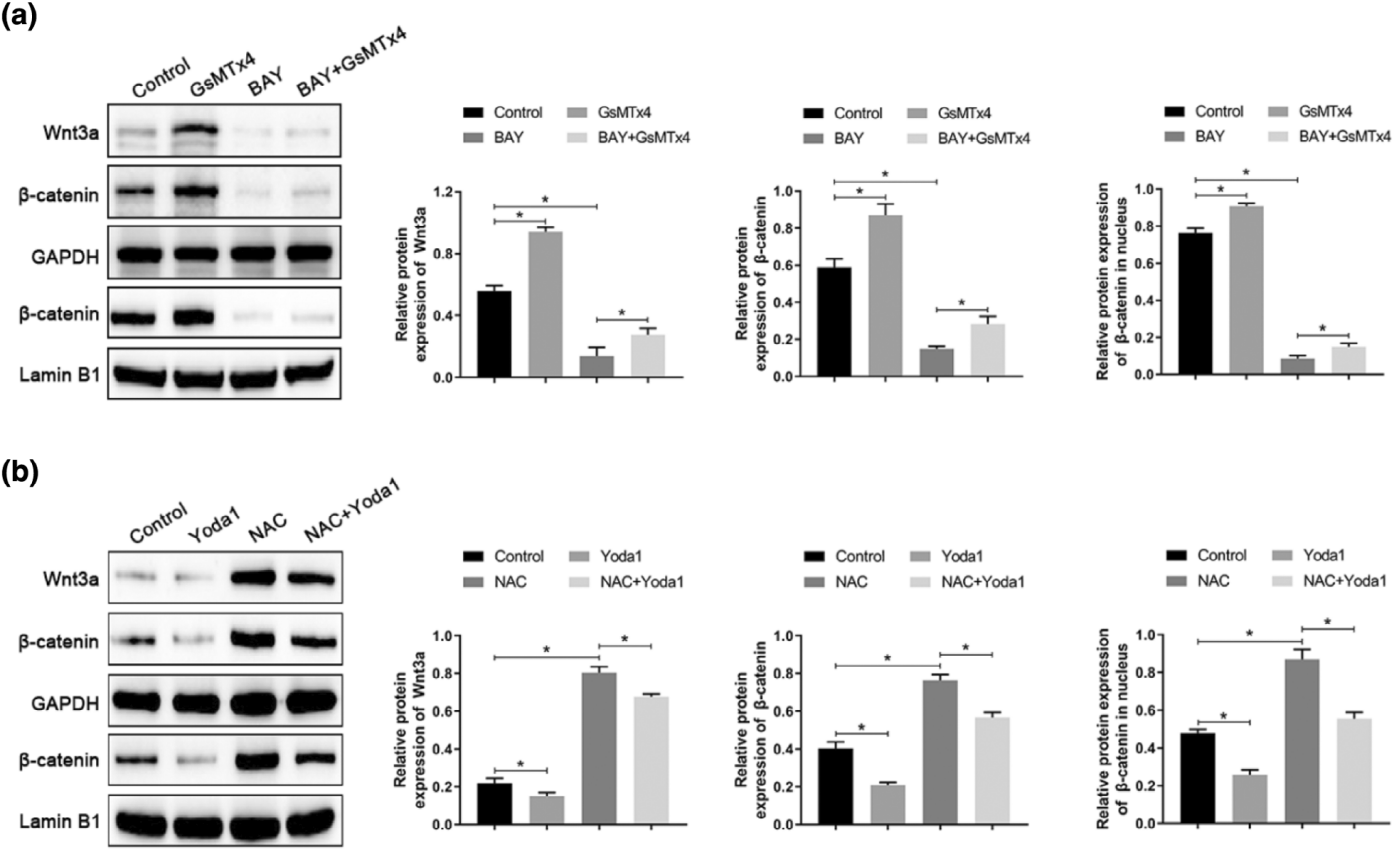
PIEZO1 regulates the activation of the Wnt/β‐catenin pathway by regulating ROS levels. (a) The effects of GsMTx4 and BAY 87‐2243 on activation of the Wnt/β‐catenin pathway in A549 cells were detected by western blotting analysis. (b) The effects of Yoda1 and NAC on the activation of the Wnt/β‐catenin pathway in A549 cells were detected by western blotting. Data are presented as the mean ± standard deviation (SD). *p < 0.05. ROS, reactive oxygen species.

ROS can stimulate the Wnt/β‐catenin pathway in several cellular processes. To clarify whether the effect of PIEZO1 on the Wnt/β‐catenin pathway is mediated via ROS, we treated A549 cells with GsMTx4 (PIEZO1 inhibitor), Yoda1 (PIEZO1 agonist), NAC (ROS inhibitor), and BAY 87‐2243 (ROS agonist), respectively. The expression of Wnt3a and β‐catenin in A549 cells and β‐catenin in the nucleus were significantly decreased after treatment with BAY 87‐2243. Treatment with GsMTx4 could reverse the inhibitory effect of BAY 87‐2243 on the activation of the Wnt/β‐catenin pathway (Figure 4a). In addition, NAC exerted an effect opposite to that of BAY 87‐2243 in promoting Wnt/β‐catenin pathway activation in A549 cells, and treatment with Yoda1 could reverse the NAC‐mediated promoting effect on Wnt/β‐catenin pathway activation (Figure 4b). Taken together, these results suggest that PIEZO1 can impact the activation of the Wnt/β‐catenin pathway by regulating ROS levels.

### PIEZO1 influences EMT processes by regulating the Wnt/β‐catenin pathway in A549 cells

**FIGURE 5:**
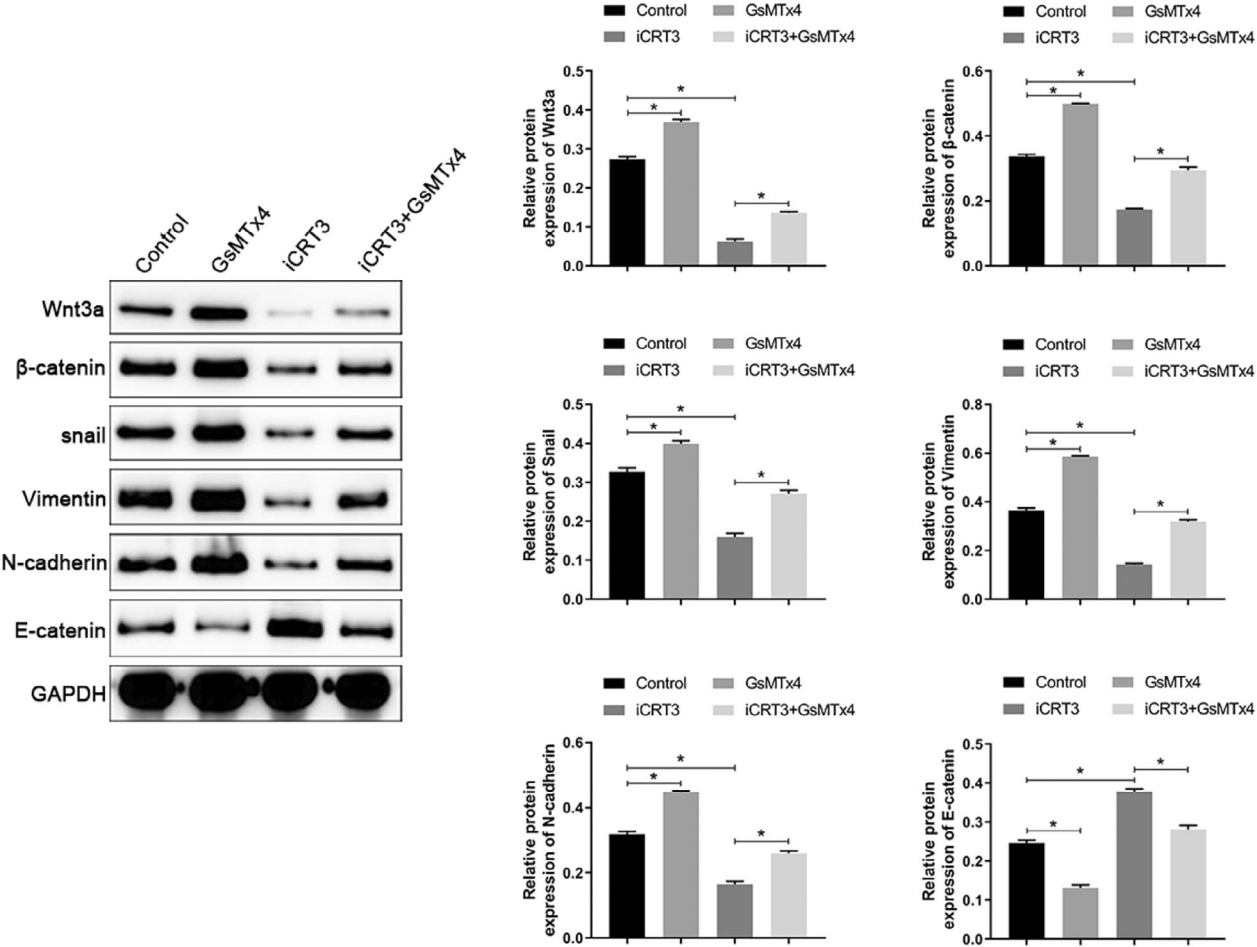
PIEZO1 regulates the Wnt/β‐catenin pathway in A549 cells to influence EMT processes. Data are presented as the mean ± standard deviation (SD). *p < 0.05. EMT, epithelial‐mesenchymal transition.

**FIGURE 6:**
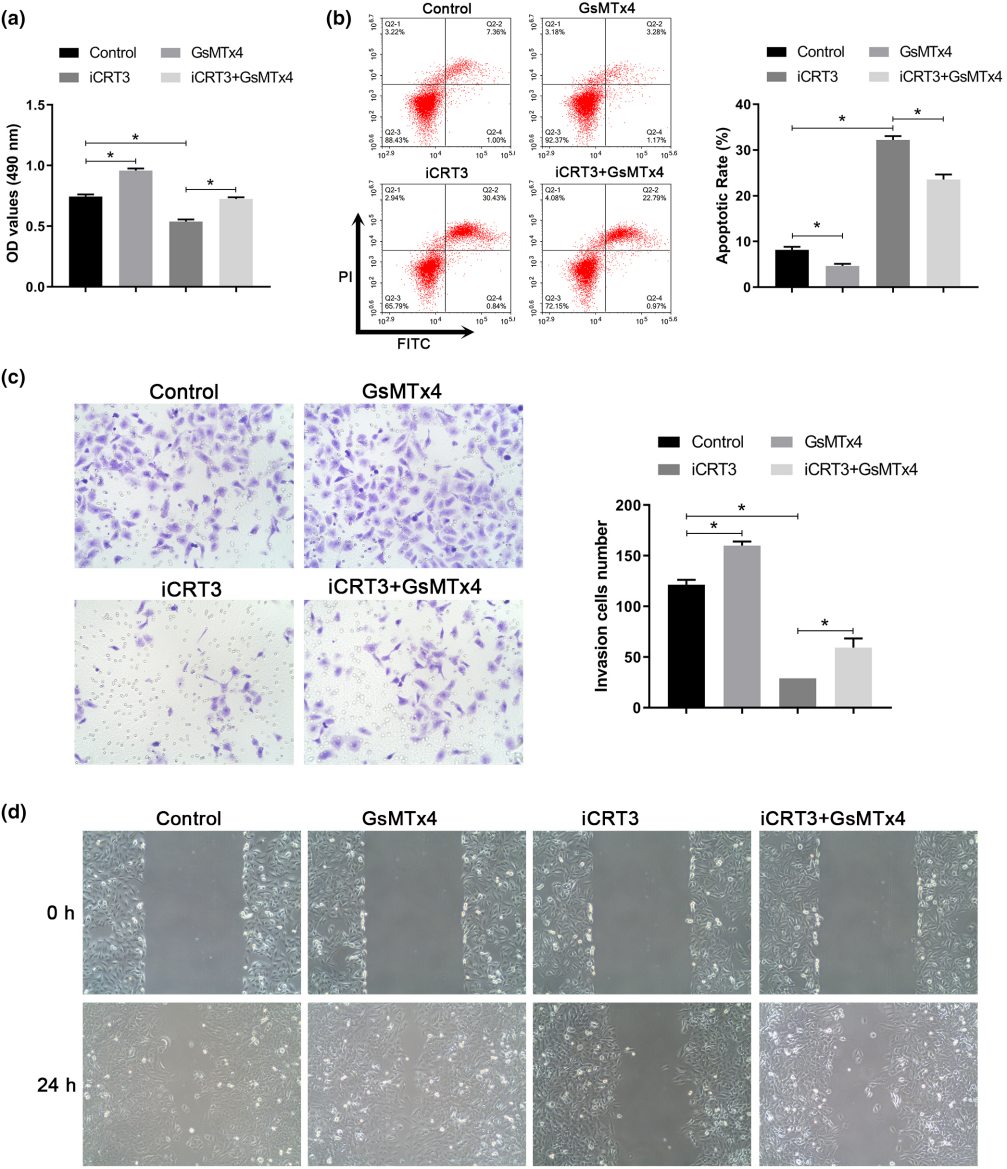
PIEZO1 influences cell proliferation, apoptosis, invasion, and migration by regulating the Wnt/β‐catenin pathway in A549 cells. (a) The cell viability of A549 cells was detected by MTT assay. (b) The apoptosis rate of A549 cells was detected by flow cytometry. (c) Cell invasion was evaluated using the transwell assay (×200). (d) Cell migration was evaluated by performing the wound‐healing assay (×100). Data are presented as the mean ± standard deviation (SD). *p < 0.05.

PIEZO1 has been shown to influence the activation of the Wnt/β‐catenin pathway by regulating ROS levels in A549 cells, and EMT is known to play an important role in lung cancer progression. To clarify the effect of PIEZO1 on the EMT process in A549 cells via the Wnt/β‐catenin pathway, we treated A549 cells with the GsMTx4 (PIEZO1 inhibitor) and the iCRT3 (Wnt/β‐catenin pathway inhibitor), respectively. We found that treatment with GsMTx4 reversed the inhibitory effect of iCRT3 on Wnt/β‐catenin pathway activation in A549 cells. Western blotting confirmed that iCRT3 inhibited the expression of Snail, vimentin, and N‐cadherin and promoted E‐cadherin expression, whereas treatment with GsMTx4 could reverse these effects (Figure 5). Finally, we examined the effects of PIEZO1 on the proliferation, apoptosis, invasion, and migration of A549 cells through the Wnt/β‐catenin pathway. Treatment with iCRT3 inhibited the proliferation, invasion, and migration of A549 cells, as well as suppressed apoptosis. Treatment with GsMTx4 reversed the effects of iCRT3 on A549 cell proliferation, apoptosis, invasion, and migration (Figure 6). These results suggest that PIEZO1 influences the biological behavior and EMT process by regulating the Wnt/β‐catenin pathway.

## DISCUSSION

In the current study, we revealed the crucial role of PIEZO1 in regulating the biological behavior and EMT of LUAD cells. PIEZO1 could affect cell proliferation, invasion, migration, and apoptosis by regulating the expression of ROS‐ and EMT‐related proteins. In A549 cells, low levels of PIEZO1 could suppress ROS levels, promote EMT processes, and facilitate cell proliferation, migration, and invasion by regulating the Wnt/β‐catenin signaling pathway.

In oral squamous cell carcinoma cells, PIEZO1 is reportedly involved in regulating the proliferation and growth of oral squamous cell carcinoma cells as a transcriptional target of YAP signaling.^21^ In aggressive gliomas, PIEZO1 is highly expressed and negatively correlated with patient survival.^22^ Furthermore, PIEZO1 knockdown could inhibit the growth of glioblastoma stem cells, suppress tumor progression, and prolong survival in mice.^22^ During the pathogenesis of disc degeneration, a stiff extracellular matrix was found to activate PIEZO1 channels and enhance intracellular Ca^2+^ levels in the nucleus pulposus. Furthermore, PIEZO1 activation reportedly increases intracellular ROS levels, promotes GDP78 and CHOP expression, and exacerbates oxidative and endoplasmic reticulum stress. In contrast, PIEZO1 knockdown attenuated extracellular matrix‐induced myeloid senescence and apoptosis.^23^ Herein, we used specific PIEZO1 inhibitors and agonists to alter PIEZO1 expression in LUAD cells. Based on MTT assay and flow cytometry results, treatment of LUAD cells with GsMTx4 increased cell proliferative activity and decreased apoptosis. Furthermore, we found that treatment with GsMTx4 induced the invasive and migratory abilities of LUAD cells, and these effects were supported by upregulating the expression of Snail, vimentin, and N‐cadherin and downregulating that of E‐cadherin. Our preliminary studies suggest that PIEZO1 plays a role in inhibiting cell proliferation, migration, invasion, and EMT during the development of LUAD in vitro, consistent with the findings of Huang et al.^13^

ROS play a crucial role in the pathogenesis of several human diseases, including metabolic disorders, neurodegenerative diseases, inflammatory diseases, tumors, and pathologies of the heart, kidneys, and lungs.^24^, ^25^, ^26^ ROS are regulatory molecules of cell signaling and gene expression involved in cell growth, survival, apoptosis, migration, and death.^27^ It has been reported that PIEZO1 converts the mechanical stretch of cardiomyocytes into Ca^2+^ and ROS signaling and affects cardiomyopathy progression.^28^ In addition, ROS participates in Wnt/β‐catenin pathway transduction in several cellular processes, and Ca^2+^‐mediated ROS metabolic signaling can be mediated by modulating Wnt/β‐catenin signaling output.^29^ Based on our findings, treatment with BAY 87‐2243 could enhance intracellular ROS levels, suppress A549 cell proliferation, and promote apoptosis by regulating the expression of apoptosis‐related proteins, whereas treatment with GsMTx4 could reverse these BAY 87‐2243‐mediated effects. These results suggested that PIEZO1 could impact the proliferation and apoptosis of A549 cells by regulating ROS levels. Furthermore, compared with BAY 87‐2243 alone, we observed that combined treatment with GsMTx4 increased the expression level of Wnt3a and β‐catenin and the nuclear translocation of β‐catenin in A549 cells. Compared with NAC alone, treatment with Yoda1 decreased the expression of Wnt3a and β‐catenin and the nuclear translocation of β‐catenin in A549 cells. These findings suggest that PIEZO1 influences the proliferation and apoptosis of A549 cells via the ROS/Wnt/β‐catenin axis.

EMT is considered a prerequisite for the initial tumor cells to become active and aggressive, leading to metastasis and recurrence in several cancers.^30^, ^31^ In numerous cancers, the Wnt signaling pathway is aberrantly activated and plays an important role in the EMT process.^32^ In colorectal cancer, TM4SF1 was found to promote cell metastasis and maintain the EMT phenotype and cancer stemness via the Wnt/β‐catenin/c‐Myc/SOX2 pathway.^33^ In addition, RNF115 may exert biological function in LUAD by mediating APC ubiquitination to regulate the Wnt/β‐catenin signaling pathway.^34^ Huang et al. have shown that SOX9 could promote migration, invasion, and EMT processes in NSCLC via the Wnt/β‐catenin pathway.^35^ Western blot results revealed that GsMTx4 could reverse the expression of Wnt3a and β‐catenin in A549 cells inhibited by iCRT3. Moreover, treatment with GsMTx4 reversed the effects of iCRT3 on the proliferation, apoptosis, invasion, and migration of A549 cells. In addition, iCRT3 inhibited the expression of Snail, vimentin, and N‐cadherin and promoted the expression of E‐cadherin, whereas GsMTx4 could reverse the effect of iCRT3 on the expression of EMT‐related proteins in A549 cells, thereby promoting EMT. Accordingly, these results suggest that low levels of PIEZO1 may promote EMT in A549 cells by activating the Wnt/β‐catenin pathway.

The project has the following limitations: (1) The expression level of PIEZO1 in LUAD tissue and adjacent tissue samples was not analyzed; (2) In this study, only pharmacological inhibitors and agonists were used to investigate the effects of PIEZO1 on the biological behavior of LUAD cells, and no gene knockdown or overexpression was used to confirm the results of inhibitors and agonists. (3) All experiments in this study were performed on cell lines, and the cell line results should also be verified in xenograft model in mice.

In conclusion, PIEZO1 negatively regulates LUAD cell survival. PIEZO1 influences the proliferation, apoptosis, migration, invasion, and EMT of A549 cells via the ROS/Wnt/β‐catenin axis. Therefore, targeted activation of PIEZO1 protein expression could be explored in treating LUAD.

## AUTHOR CONTRIBUTIONS

Haimei Bo and Qi Wu designed and conducted the study, performed a literature search, undertook the statistical analysis, and prepared the figures. Chaonan Zhu, Yang Zheng, Guang Cheng, and Lihua Cui assisted with data collection, analysis, and interpretation. Haimei Bo and Qi Wu drafted and revised the manuscript. All authors read and approved the final manuscript before submission.

## CONFLICT OF INTEREST STATEMENT

The authors confirm there are no conflicts of interest.

## Data Availability

Data used to support the findings of this study are available from the corresponding author upon request.
